# Re-examining the relationship between audiometric profile and tinnitus pitch

**DOI:** 10.3109/14992027.2010.551221

**Published:** 2011-03-09

**Authors:** Magdalena Sereda, Deborah A Hall, Daniel J Bosnyak, Mark Edmondson-Jones, Larry E Roberts, Peyman Adjamian, Alan R Palmer

**Affiliations:** * MRC Institute of Hearing Research, University Park, Nottingham, UK; † NIHR National Biomedical Research Unit in Hearing, Nottingham, UK; ‡ Division of Psychology, School of Social Sciences, Nottingham Trent University, Nottingham; § Department of Psychology, Neuroscience, and Behavior, McMaster University, Hamilton, Ontario, Canada

**Keywords:** Audiogram, Audiometric edge, Correlation, Principal components, Multiple regression

## Abstract

*Objective:* We explored the relationship between audiogram shape and tinnitus pitch to answer questions arising from neurophysiological models of tinnitus: ‘Is the dominant tinnitus pitch associated with the edge of hearing loss?’ and ‘Is such a relationship more robust in people with narrow tinnitus bandwidth or steep sloping hearing loss?’ *Design:* A broken-stick fitting objectively quantified slope, degree and edge of hearing loss up to 16 kHz. Tinnitus pitch was characterized up to 12 kHz. We used correlation and multiple regression analyses for examining relationships with many potentially predictive audiometric variables. *Study Sample:* 67 people with chronic bilateral tinnitus (43 men and 24 women, aged from 22 to 81 years). *Results:* In this ample of 67 subjects correlation failed to reveal any relationship between the tinnitus pitch and the edge frequency. The tinnitus pitch generally fell within the area of hearing loss. The pitch of the tinnitus in a subset of subjects with a narrow tinnitus bandwidth (n = 23) was associated with the audiometric edge. *Conclusions:* Our findings concerning subjects with narrow tinnitus bandwidth suggest that this can be used as an *a priori* inclusion criterion. A large group of such subjects should be tested to confirm these results.

## Abstract (sp)

*Objetivo:* Exploramos la relación entre la forma del audiograma y el tono del acufeno para responder a preguntas provenientes de modelos neurofisiológicos del acúfeno: ‘Es el tono dominante del acúfeno asociado con el borde de la hipoacusia?'y ‘Será tal relación más robusta en personas con un acúfeno de banda angosta o con una hipoacusia de caída abrupta?’ *Diseño:* Una pendiente tipo “palo roto” objetivamente cuantificada, con severidad y borde de la hipoacusia hasta 16 kHz. El tono del acúfeno fue caracterizado hasta 12 kHz. Utiliza-mos un análisis de correlación y regresión múltiple para examinar la relación con muchas de las variables potencialmente predictivas. *Muestra del Estudio:* 67 personas con acúfeno bilateral crónico (43 hombres y 24 mujeres con edades entre 22 y 81 años). *Resultados:* En esta amplia muestra de 67 sujetos, la correlación no demostró ninguna relación entre el acúfeno y el borde de la frecuencia. El tono del acúfeno generalmente cayó dentro del área de la hipoacusia. En un subgrupo de sujetos, que tenían un acúfeno de banda angosta (n=23), si hubo asociación con el borde audiométrico. *Conclusiones:* Nuestros hallazgos que corresponden a los sujetos con un acúfeno de banda angosta, sugieren que esto puede ser utilizado a *priori* como un criterio de inclusión. Un grupo grande de sujetos debe ser examinado para confirmar estos resultados.

The prevalence of tinnitus is high. In the general population, it is estimated at around 10–15% (Andersson et al, 2005), while for patients attending otology clinics it is estimated that 70–90% experience tinnitus either as the main or associated symptom (Andersson et al, 2005). In many cases, tinnitus is associated with some kind of auditory impairment, especially high-frequency sensorineural hearing loss (Jacobson et al, 1969; Vernon et al, 1980; Barnea et al, 1990). In this paper, we examine the relationship between properties of the hearing impairment and the frequencies that make up tinnitus, which has been a topic of recent debate.

Recent neurophysiological models of tinnitus predict one of two relationships between the audiometric profile and the dominant tinnitus pitch. The ‘tonotopic expansion’ model suggests that tinnitus is the perceptual consequence of a plastic transformation of the orderly representation of single frequencies within central auditory structures (see Rauschecker, 1999 for a review). Loss or reduction in cochlear inputs to a frequency-specific region of the tonotopic map results in a reorganization whereby the frequency tuning properties of neurons above the audiometric edge shift down towards the lowest frequency that receives ‘normal’ input (Robertson & Irvine, 1989; Harrison et al, 1991; Rajan et al, 1993; Eggermont & Komiya, 2000). As a result, frequencies at the edge of the hearing impairment become over-represented in the tonotopic map, and it has been suggested that this gives rise to the tinnitus percept (see Eggermont & Roberts, 2004). A second group of models (e.g. Kiang et al, 1969; Llinás et al, 2005) attributes tinnitus to a contrast enhancement of neural activity spanning the audiometric edge similar to that observed in other sensory systems at the borders of discontinuities of excitation and inhibition in sensory maps. Like the tonotopic expansion model, contrast enhancement models propose that tinnitus can be characterized by a dominant frequency (the tinnitus pitch) localizing close to the edge of normal hearing. In comparison with these views, the ‘neural synchrony’ model suggests that tinnitus is generated by spontaneous synchronous neural activity that develops among hyperactive neurons in the hearing loss region (Eggermont & Roberts, 2004). The output of these neurons is heard in terms of their original cochleotopic tuning and gives the tinnitus percept, that would correspond to somewhere within the region of hearing loss more often than to the audiometric edge. While demonstrating a correspondence between the tinnitus pitch and the edge of the hearing loss would support tonotopic expansion and edge frequency models, demonstrating that tinnitus is more closely associated with the region of hearing impairment is consistent with a number of physiological explanations including increased neural synchrony and hyperactivity in the hearing loss region.

Current findings regarding the relationship of the audiometric profile to tinnitus frequencies in human participants are somewhat mixed. If one looks in the literature, some claim a systematic relationship between pitch and the audiometric edge, while others disagree, and the remainder report a less specific relationship between tinnitus pitch and the region of hearing loss. At least four studies have found no overall systematic relationship between the most prominent tinnitus pitch and the shape of hearing loss (Tyler & Conrad-Armes, 1984; Meikle, 1995; Tyler, 2000; Pan et al, 2009). However, similar numbers of studies report positive results. Penner (1980) reported four patients in whom the pitch-matched frequency was just below the edge of a precipitous high-frequency noise-induced hearing loss. This observation may align with the report of Rajan and Irvine (1996) who found that only in cases where the hearing loss was steeply sloping (at least 50 dB per octave) was there clear evidence for tonotopic expansion, as evidenced by changes in synaptic connectivity. Similarly, Hazell and Jastreboff (1990) commented that many tinnitus patients match their tinnitus pitch to the frequency close to the edge of hearing loss. More recently, König et al (2006) found an association between tinnitus pitch and edge frequency in a subgroup of 24 patients with noise-induced hearing loss who reported that their tinnitus was tone-like, but not in the subgroup of 17 patients who reported tinnitus with broad spectral features. The study by Pan et al (2009) had greater statistical power because they examined a large cohort of 195 patients. Nevertheless, they failed to find a relationship between tinnitus pitch and the edge of hearing loss or two other measures of hearing loss (average of the hearing thresholds across frequencies and frequency of the maximum hearing loss). These authors noted that *some* individuals did exhibit a pitch at the low-frequency edge of the hearing loss. From these observations, they postulated that a relationship between pitch and audiometric profile might be present in a certain subgroup of people with tinnitus. However, they were unable to identify what might be the important defining characteristics. A number of authors highlight their finding that a majority of people with tinnitus report a dominant tinnitus pitch that falls within the area of hearing loss (Henry & Meikle, 1999; Noreña et al, 2002; Pan et al, 2009) or a percept that spans a broad spectrum, also co-located with the area of hearing loss (Noreña et al, 2002; Roberts et al, 2006, 2008).

In this paper we undertake analyses of existing data and new data collection in an attempt to reconcile findings regarding the relation of tinnitus sounds to hearing impairment. We apply a careful methodology to address four of the main challenges that have so far limited the extent to which the present findings can be interpreted; (1) we specify objective criteria for quantifying the shape of the audiogram and we consider a larger part of the audible range (up to 16 kHz), (2) we use a psychophysical method to estimate tinnitus pitch in a way that does not rely on obtaining a pitch match to a single-frequency and that attempts to separate loudness and pitch similarity ratings, (3) we apply a rigorous statistical method for testing the relationships, and (4) we try to account for the heterogeneity of tinnitus by using two *planned* criteria for selecting particular subgroups of the cohort. We briefly expand on these four points in turn, highlighting some of the implications for interpreting previous results and our strategies for addressing them in our present methodology.

While the pure-tone audiogram indicates portions of the basi-lar membrane with reduced sensitivity for detecting sound energy at low levels, it does not determining the physical integrity of the inner and outer hair cells in those regions (Halpin & Rauch, 2009). Nevertheless, any plasticity in frequency representation is likely be driven by this functional loss and so audiometry is still informative. Clearly defined criteria for quantifying the shape of the audiogram are crucial for exploring its relationship with tinnitus pitch. Different studies have adopted different criteria for determining the frequency that corresponds to the audiometric edge. The lack of a standardized procedure makes study outcomes hard to interpret and compare. Several examples are provided for illustration. König et al (2006) used a two-step procedure that applied a linear curve fitting algorithm to the audiogram. First, they identified the range of frequencies for which hearing levels do not drop more than 20 dB below the best hearing level and replotted these data according to the steepness of the hearing loss across frequencies (second derivative). Within this frequency range, the audiometric edge was identified by the maximum of the second derivative of the audiogram. Pan et al (2009) also used a two-step procedure that considered the frequency differences between series of adjacent frequencies by visually inspecting the audiogram. First, pairs of frequencies were considered and the lowest frequency pair whose difference exceeded 15 dB was selected. The audiometric edge was defined as the lower of these two frequencies. If that condition was not satisfied, then the lowest adjacent three frequencies whose difference exceeded 25 dB were selected. In that case, the audiometric edge was defined as the lowest of these three frequencies. In both examples, the audiometric edge was constrained to correspond to one of the octave intervals measured in the audiogram. For quantifying audiometric variables in an objective manner that is independent of the specific frequencies tested, we propose a novel application of a well-established curve-fitting procedure (broken stick) that provides measures of the audiometric edge, the slope, and the degree of hearing loss.

A second challenge in interpreting current findings concerns the method that has been used to estimate the dominant tinnitus pitch, as described above. Tinnitus pitch is most often estimated using a pitch-matching procedure whereby an external sound (usually a single-frequency tone) is selected that best matches the dominant tinnitus pitch (Vernon & Meikle, 1981; Meikle, 1995; Noreña et al, 2002; see also Henry & Meikle, 2000 for a review). However, this method has rather poor test-retest reliability (Penner, 1983; Henry & Meikle, 2000). The choice of match is also susceptible to adaptation by the external tone which might influence the perception of that tone, or is susceptible to time-varying dependencies when the tinnitus percept is fluctuating (Vernon & Meikle, 1988). Despite the fact that normal listeners appear able to match a pure tone to a complex tone (Peters et al, 1982), the main criticism of this test is that tinnitus patients may find it difficult to choose a single frequency that best describes their tinnitus, especially when their tinnitus is not tonal. In recent years, some authors have started to assess tinnitus pitch by asking people to rate the ‘contribution’ (Noreña et al, 2002) or ‘likeness’ (Roberts et al, 2006; 2008) of individual frequencies within the audiometric range to the overall tinnitus percept. It is possible that when one frequency in a tinnitus spectrum is judged to be clearly dominant over other frequencies, it may correspond to the tinnitus pitch. A study that has directly compared the two methods and found that, while the two procedures yielded somewhat similar estimates of the dominant tinnitus pitch, participants preferred the ‘likeness’ approach because it did not challenge them with uncomfortable choices (Kay & Searchfield, 2008). Moreover, the study by Moffat et al (2009) demonstrated that tinnitus spectra measures were stable over time, indicating acceptable test-retest reliability of that method. The ‘likeness’ procedure is the method used in the present study.

The third limitation concerns the statistical methods applied to examine the relationship between the shape of hearing loss and the tinnitus pitch. Studies to date have typically examined this relationship using correlation statistics (e.g. a Pearson two-tailed correlation) (König et al, 2006; Pan et al, 2009). For example, Pan et al (2009) assessed the degree of correlation between the reported tinnitus pitch and three audiometric variables (edge of hearing loss, pure-tone average, and maximum hearing-loss frequency) for 34 different subcategories of the patient cohort. The exploratory nature of the investigation should have lead to conservative statistical testing. Without a correction of the probability threshold to account for multiple comparisons, *post-hoc* testing inflates the likelihood of a false positive result. Here we report adjusted significance levels where appropriate and also the corresponding confidence intervals for r, which define the likely range of the true value in the population from which the sample was drawn. Equally important, correlational statistics do not lend themselves to accurately characterizing complex relationships when two or more of the audiometric variables are correlated with one another and when two or more audiometric variables contribute to the tinnitus pitch. While we report correlation statistics for comparison with previous studies, we also apply principal component analysis and multiple regression. These methods overcome the problem of colinearity between audiometric variables and the problem of multiple comparisons.

Given the heterogeneity of tinnitus, most authors would agree that it is probably inappropriate to pool all tinnitus participants into a single group for statistical analysis. In the present study, we therefore not only investigate potential relationships across the whole cohort, but we specifically test our hypotheses in subgroups of patients chosen according to a narrow tinnitus bandwidth (c.f. König et al, 2006) and a steep slope of hearing loss (Rajan & Irvine, 1996).

## Methods

### Participants

Audiometry and tinnitus data were collected from 67 volunteers with bilateral tinnitus (43 men and 24 women, aged from 22 to 81 years) tested at one of three research sites. These sites were McMaster University in Hamilton, Canada (47 subjects), the University of British Columbia in Vancouver, Canada (12 subjects) and MRC Institute of Hearing Research, Nottingham, UK (8 subjects). Subjects were recruited from the ENT clinic at McMaster University Medical Center (Hamilton cohort), ENT clinics at hospitals affiliated with the University of British Columbia (Vancouver cohort), Nottingham Audiology Services (Nottingham cohort), as well as advertisements in the local newspapers (Hamilton and Vancouver cohorts). Out of the cohort, 40 people reported tonal tinnitus, 15 people reported ringing tinnitus, and 12 people had hissing tinnitus (see later for definitions). For most subjects (N = 55), their tinnitus was steady over time, although some (N = 12) reported a pulsing sensation.

### Testing procedures

**Figure 1:**
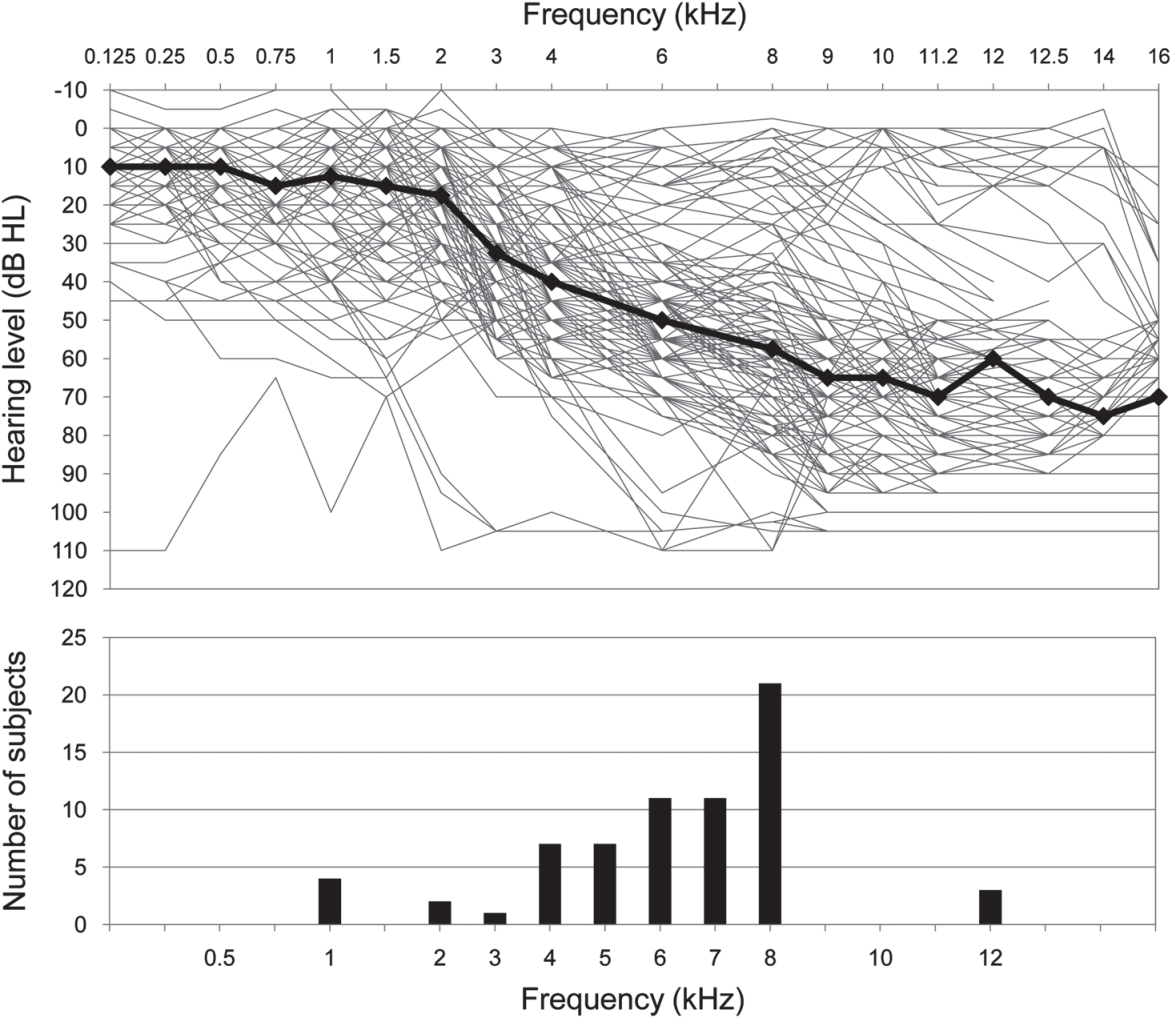
Association between hearing level and the dominant tinnitus pitch. Top panel illustrates audiometric thresholds for all 67 patients (134 ears) with the median data shown by the black line. Bottom panel shows the distribution of the dominant tinnitus pitch derived from the similarity ratings.

Hearing levels for the two ears were measured up to 16 kHz (Hamilton cohort) or 12 kHz (Vancouver and Nottingham cohorts) using standard audiometric procedures (see Figure 1). An automated, computerized procedure, the Tinnitus Tester (Roberts et al, 2006, 2008), was used to assess the psychoacoustical properties of the tinnitus in each patient. Initially, a familiarization programme introduced participants to the graphical user interface, as well as to the concepts of loudness and pitch. The test battery assessed the laterality of the tinnitus sensation (i.e. left, right, or bilateral), classified its spectral properties (i.e. tonal, ringing, or hissing), its temporal properties (i.e. ‘steady’ or ‘pulsing') and also measured its loudness and frequency spectrum. A previous study (Roberts et al, 2008) had been unable to demonstrate any significant correlation between the temporal characteristics of the tinnitus and the shape of the audiogram or the tinnitus spectrum, and so temporal properties were not considered further. Spectral properties were classified by asking participants to select one of three sounds that best characterized their tinnitus (see Roberts et al, 2008). For ‘tonal’ tinnitus the sound was a 5-kHz pure tone, for ‘ringing’ tinnitus it was a bandpassed noise whose spectrum was ± 5% of the 5-kHz centre frequency, and for ‘hissing’ tinnitus it was a bandpassed noise at ± 15% of the 5-kHz centre frequency, each measured at 10 dB below the spectral peak. The three types of sounds are clearly discriminable and patients did not seem to experience any problem indicating which sound best characterized the spectral quality of their tinnitus.

The choice of tinnitus spectral property determined the bandwidth of the target frequencies in the loudness and frequency stages of the test battery. Loudness was first quantified using a visual-analogue rating scale, and second by asking people to adjust the level of each frequency (in dB SPL) so that it matched the loudness of their tinnitus. Eleven different centre frequencies (from 0.5 kHz to 12.0 kHz) were presented and the adjustment was capped at a maximum of 95 dB SPL. The frequency spectrum was quantified by asking people to indicate the similarity of their tinnitus to each presented frequency. Each of the frequencies was presented three times and the mean value of three judgements was taken to represent the similarity value for that frequency. In this phase the tones were presented at the same loudness that was chosen to match their tinnitus loudness in the previous phase. Loudness and pitch ratings were performed using a Borg CR100 scale (Borg & Borg, 2001; i.e. 0 = not at all, 30 = not very similar, 50 = somewhat similar, 70 = very similar, 100 = identical). The Borg scale is commonly used to measure subjective perceptions and along with the visual analogue scale has been shown to have good reproducibility (Capodaglio, 2001). Loudness measures were not analysed in the present study.

### Quantification of the audiometric data

**Figure 2:**
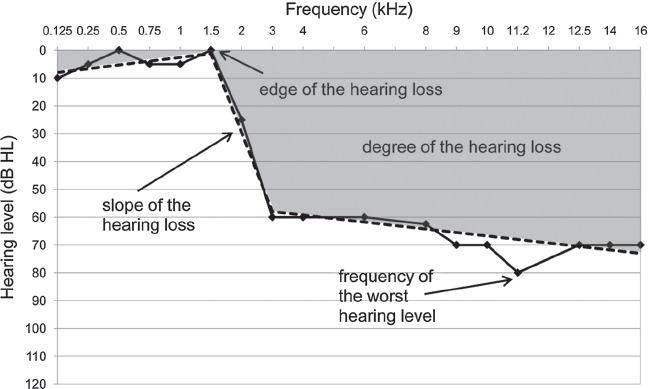
An example of the ‘broken-stick’ function fi tted to the audiometric data. The solid line shows individual patient's hearing level and the broken line shows non-linear regression with two breaks (the best fit for that hearing profile). The ‘broken stick’ function was used to quantify the audiometric edge, slope, degree of hearing loss (shaded area). Frequency of the worst hearing level was also identifi ed.

The audiometric profile was used to quantify a number of different aspects of the hearing loss; namely the audiometric edge, slope, degree of hearing loss, frequency of the worst hearing level, and the ear with the steeper hearing loss (Figure 2). To improve the estimates of these parameters, any noise in the audiometric data was reduced by fitting a function to the observed values. Three different functions were fit to the individual hearing levels using a bespoke Matlab procedure. One of the functions was a simple linear regression (0-break), the other two were non-linear ‘broken stick’ regressions with one break or two breaks. For the audiograms measured up to 12 kHz, the regression fits were extrapolated up to 16 kHz. We accept that for these cases we may have failed to detect an edge above 12 kHz. The best fitting broken-stick function was assessed using the following parametric bootstrap approach. Based on the 0-break function, new data points were simulated with normally distributed errors. The procedure then evaluated the degree to which the 1-break solution improved the goodness of fit over the 0-break solution using a least-squares comparison. In cases where the 1 -break solution was better, the same approach was then used to compare it with the 2-break solution.

#### Edge of the hearing loss

The frequency at which the break of the function occurred was taken as the edge of the hearing loss. In the case of 1- and 2-break fits, the edge was constrained to correspond to the break point of the portion of the regression line that passed from clinically normal (<20 dB HL) to impaired (>20 dB HL; see Figure 2). In the ears where a simple linear regression best described the audiogram, there was no edge of hearing loss.

#### Slope of the hearing loss

The slope of the regression function represented the slope of the hearing loss in each ear, calculated in dB/octave. In the case of the 1- or 2-break solutions, the slope was taken as the portion of the regression line that occurred directly after the edge of the hearing loss. For all analyses, the slope of the hearing loss was used as a categorical variable for investigating effects of the other audiometric and tinnitus variables according to the ‘steeper’ and in the ‘less-steep’ ear. Thus we defined two groups of 67 observations; one group representing the ears with a steeper slope of hearing loss and another representing all ears with a less steep slope. Data for each patient appeared once in each group. For the ‘steeper’ ear, the slope was used as a further categorical variable to split the dataset into thirds: a group with a steep slope (36.5-214.0 dB/octave), a group with a moderate slope (20.5-36.4 dB/octave), and a group with a shallow slope (4.0-20.4 dB/octave).

#### Degree of hearing loss

A standardized clinical procedure defines the degree of hearing loss as the mean hearing level across 0.25, 0.5, 1, 2, 4 kHz (British Society of Audiology guidelines, 2004). This method fails to account for higher frequencies which might be relevant for tinnitus generation (Jacobson et al, 1969; Barnea et al, 1990). In the present study, the degree of hearing loss was represented by the area underneath the curve from 0.125 to 16 kHz and from 0 to 120 dB HL, calculated in dB*octave. In some cases, hearing at some frequencies was more sensitive than 0 dB HL, and so this area was subtracted from the total area of the hearing loss.

#### Frequency of the worst hearing level

This variable was the frequency at which the hearing loss reached a maximum value. Whenever there were two or more frequencies with the maximum loss, the lowest frequency was recorded for further analysis.

### Quantification of the tinnitus data

#### Dominant pitch

The dominant pitch was taken from the pitch-similarity ratings (i.e. the frequency that was rated as the most similar to the tinnitus pitch). Three of 67 participants rated more than one frequency equally to be ‘most like’ their tinnitus. In these cases, we selected the frequency closest to the edge of the hearing loss to represent the dominant tinnitus pitch. One rationale for selecting the frequency closest to the edge was that this choice represents the ‘best’ evidence to support the theory of tinnitus as an over-representation of the lesion edge in the tonotopic map. If the subsequent analysis failed to support a relationship between tinnitus pitch and edge frequency then this could be due to a bias in our data selection. Secondly, when several tones were rated as ‘most like’ the tinnitus they were often far apart on the frequency axis. In these cases, the alternative geometric mean estimate would seem inappropriate.

#### Bandwidth

The width of the tinnitus spectrum was also derived from the pitch-similarity ratings. To derive a single measure of bandwidth that was independent of the obtained similarity scores, bandwidth was calculated as the standard deviation of the weighted frequencies, where large weights were given to those frequencies rated as most similar to the tinnitus. The Borg scale was used to assess similarity (Borg & Borg, 2001) and the values obtained were used as the weights. For some analyses, bandwidth was used as a categorical variable to split the dataset into thirds: a group with a narrow bandwidth (0.13-0.25 kHz), a group with a moderate bandwidth (0.26-0.33 kHz), and a group with a wide bandwidth (0.34-0.44 kHz).

### Exploring the relationship between audiometric profile and tinnitus

Many of the variables were not normally distributed and so these were transformed by taking a natural logarithmic transform of the values. The measure of tinnitus bandwidth satisfied a normal distribution and so was not transformed. For direct comparison with previous literature, we first report the results of simple correlation statistics. However, we interpret these results with caution, most notably because the audiometric variables (edge of the hearing loss, slope of the hearing loss, degree of hearing loss, and the frequency of the worst hearing level, for the two ears) were highly inter-correlated (see Appendix at http://www.informahealthcare.com/doi/abs/10.3109/14992027.2010.551221). In fact, 17 out of the 28 pairs were significantly correlated. While correlational analysis is usually used to explore the relationship between two variables, multiple regression analysis allows us to assess the relationship between several audiometric variables and tinnitus pitch. However, including predictor variables that are highly correlated with other predictor variables is not recommended as the new predictor variable is associated with variance which has already been explained by the model. As a more rigorous alternative strategy for examining our hypotheses, we have therefore implemented a multiple regression analysis using a set of predictor variables that are *not* intercorrelated. These variables were derived from a principal components analysis: a mathematical procedure that transforms a number of potentially correlated variables into a smaller number of uncorrelated variables.

## Results

### Audiometric data: Descriptive statistics

From the broken-stick fitting procedure that was applied to the 134 ears (67 participants), a 0-break fit best described the audiogram for 19 ears, a 1-break fit best described the audiogram for 81 ears, and a 2-break fit was chosen for 34 ears. From the 1- and 2-break solutions, our criterion for defining the edge of the hearing loss was met in the majority of cases (106 out of 115 ears). There appeared to be a large amount of inter-subject variability in the values obtained. For those ears in which an edge was identified, it ranged from 0.13 to 14.33 kHz (mean = 2.74 and SD = 2.72). Across all 134 ears, the slope of the hearing loss ranged from 0.09 to 214.38 dB/octave (mean = 28.96, SD = 29.18), and degree of hearing loss ranged from 2.46 to 105.73 dB/octave (mean = 62.38, SD = 23.03).

### Tinnitus data: Descriptive statistics

**Figure 3:**
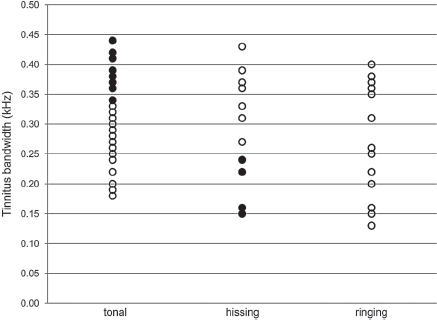
Individual ratings of the spectral properties of tinnitus plotted against subsequent numerical estimates of tinnitus bandwidth. The filled circles highlight unexpected patterns of association between these variables. Eight ofthe subjects reporting ‘tonal'tinnitus formed part of the subgroup with the widest numerical bandwidth (0.34–0.44). Similarly, four ofthe subjects reporting ‘hissing'tinnitus formed part ofthe subgroup with the narrowest numerical bandwidth (0.13–0.25). We have only used these symbols for the two extreme bandwidth groups (tonal and hissing). We have no specific *a priori* expectations for the intermediate (ringing) group.

Likewise, there was a large amount of inter-subject variability in the observed tinnitus characteristics. The dominant tinnitus pitch ranged from 0.5 to 12 kHz (mean = 6.26, SD = 2.43) and bandwidth varied from 0.13 to 0.44 (mean = 0.29, SD = 0.09). A one-way ANOVA revealed that the participant's choice about the spectral property of their tinnitus (i.e. tonal, ringing, or hissing) was unrelated to the subsequent numerical estimate of bandwidth (F [2, 64] = 0.72, p = .49). The associations between subjective classification and the bandwidth estimate are plotted in Figure 3. The expectation is that a tinnitus with a narrow range of frequency components would be judged as ‘tonal', while a ‘tinnitus with a broad frequency spectrum would be judged as ‘hissing'. Clearly some subjects reporting tonal tinnitus actually rated a wide range of frequencies to be similar to their tinnitus, while others who reported hissing tinnitus later judged a narrow range of frequencies to be similar to their tinnitus. One interpretation that concurs with previous observations is that people's subjective description is not very reliable when it comes to selecting sub-groups of subjects.

### Exploratory correlations between audiometric profile and tinnitus pitch for the whole cohort

For comparison with previous studies, a series of correlations explored the degree of association between the dominant tinnitus pitch and the audiometric variables, separately for each ear (Table 1). The results showed a relationship with the degree of hearing loss in either ear (two-tailed Pearson's test, p < .05). The interpretation of this negative correlation between the dominant tinnitus pitch and the degree of hearing loss is that the milder the hearing loss (even if not clinically meaningful), the higher the perceived pitch of the tinnitus. It is broadly compatible with the neurophysiological model that predicts the tinnitus pitch to fall within the region of hearing loss, because mild presbyacusis mostly impairs hearing at the very high frequencies. However, these correlations do not survive a correction for multiple comparisons (p < .006) indicating low confidence in the result. In support of this interpretation, the lower and upper confidence limits for the correlation coefficient (r) were rather broad (steeper ear: — .467 to — .016, and less steep ear: — .537 to — .047) indicating rather weak internal validity. Calculations of the coefficient of determination (r^2^) demonstrated that only 6.5% and 8.1% of variance in the tinnitus pitch could be accounted for by degree of hearing loss (for the steeper and less steep ear, respectively).

**Table 1:** Correlations between dominant tinnitus pitch and audiometric variables computed using data for all 67 participants. HL = hearing loss. Corrected signifi cance <.006.

Audiometric variable	Number of ears	Correlation coeffi cient	P value
Edge of HL in the steeper ear	60	.096	.467
Edge of HL in the less-steep ear	46	.074	.627
Slope of HL in the steeper ear	67	.058	.640
Slope of HL in the less-steep ear	67	− .140	.261
Degree of HL in the steeper ear	67	− .255	.038
Degree of HL in the less-steep ear	67	− .284	.020
Frequency of the worst hearing level in the steeper ear	67	− .001	.995
Frequency of the worst hearing level in the less-steep ear	67	.031	.806

### Planned correlation for participants with a narrow tinnitus bandwidth

**Figure 4:**
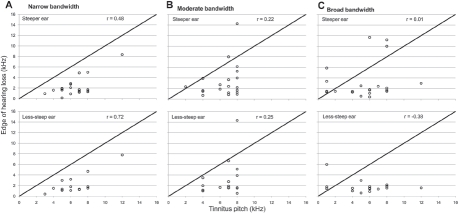
Scatterplots examining the relationship between dominant tinnitus pitch and the edge of hearing loss, as a function of the numerical bandwidth estimate of the tinnitus percept. For statistical testing, the subgroup reporting the narrow bandwidth (A) represents a planned comparison, and the subgroups with moderate (B) and broad bandwidth (C) represent *post hoc* (exploratory) comparisons. Data are presented separately for the steeper ear (top row) and the less-steep ear (bottom row).

In the Introduction, we outlined a planned comparison between the edge ofthe hearing loss and tinnitus pitch, specifically in those 23 participants who reported a narrow numerical tinnitus bandwidth (Figure 4). Significant positive relationships were found, irrespective ofthe ear in which the audiometric edge was quantified (steeper ear: r = .48, p (one-tailed) <.O5, N = 19 and less-steep ear: r = .72, p (one-tailed) <.01, N = 14; note that there was no edge frequency for 13 out ofthe total 46 ears). Although these correlation coefficients indicate a large effect, the confidence intervals were again quite broad (.032 to .767 and .310 to .906, for the steeper and less-steep ears respectively), perhaps due to the rather small sample sizes. Nevertheless, the observed data demonstrated that the estimated edge of the hearing loss accounted for 23% and 52 % ofthe variability in tinnitus pitch (for the steeper and less-steep ear data, respectively). Additional exploratory testing revealed null results for the two other subgroups (p > .1).

This result is certainly intriguing because it supports the observation reported previously by König and colleagues (König et al, 2006) that those patients experiencing a tonal tinnitus are most likely to show a relationship between audiometric edge and tinnitus pitch. This result however, does not necessarily mean that the values ofthe edge and the pitch were closely matched to one another. The edge of the hearing loss and the region ofthe hearing loss probably covary with one another. Indeed, from the individual data plotted in Figure 4A (and from König et al, 2006), it can be noted that the tinnitus pitch was rarely at the edge, but instead was typically more than an octave above the edge frequency, in the region ofthe hearing loss.

### Planned correlation for participants with a steep-sloping hearing loss

**Figure 5:**
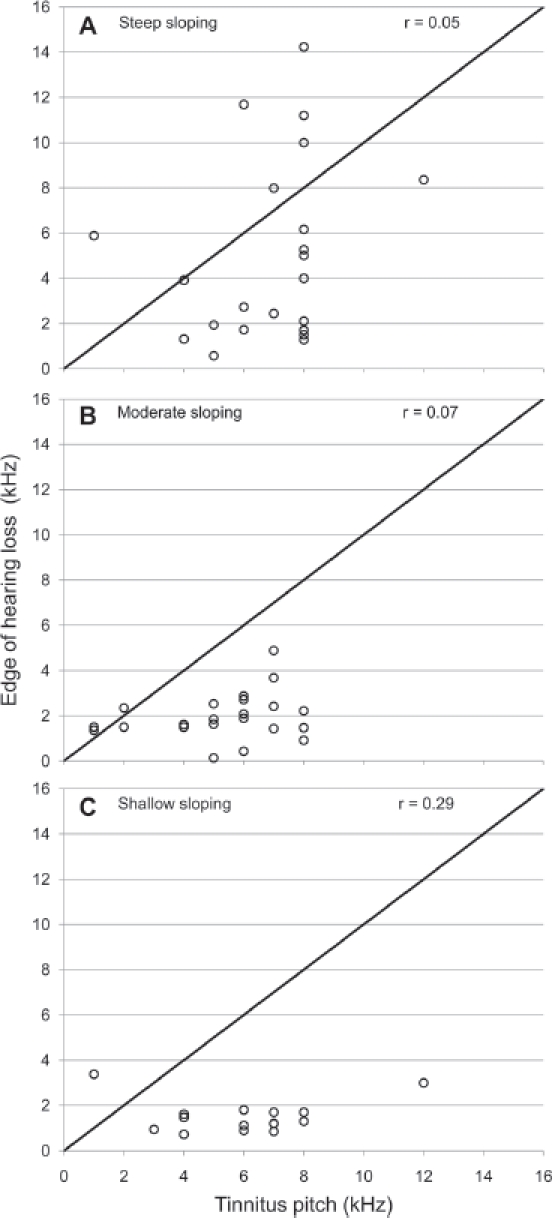
Scatterplots examining the relationship between dominant tinnitus pitch and the edge of hearing loss, as a function of the slope of hearing loss measured in the steeper ear. For statistical testing, the subgroup with the steep slope (A) represents a planned comparison and the subgroups with moderate (B) and shallow (C) slopes represent *post hoc* (exploratory) comparisons.

In the Introduction, we outlined a planned comparison between the edge of the hearing loss and tinnitus pitch in those 22 participants who had a steep-sloping hearing loss, (defined for the steeper ear). Even in this selected subject group there was no significant relationship, r = .05, p (one-tailed) < .42 (see Figure 5). Only about 0.2% ofthe variance in pitch could be explained by the edge ofthe hearing loss.

We also found null results for the other two subgroups with a moderate and shallow slopes of hearing loss (p > .3). From these findings, we conclude that the slope of hearing loss does not appear to drive individual tinnitus pitch.

### Multiple regression for the whole cohort

To generate a set of independent predictor variables describing a participant's audiometric profile, all the data were first subjected to a principal component analysis. From the original set of eight variables, eight factors were generated and these are reported in Table 2. Factors that explained at least 10% of the variance and had an eigenvalue exceeding 0.7 were carried forward to the multiple regression (Jolliffe, 1972, 1986). The three selected factors explained over 80% of the variability in the data. The first factor explained 50% of the variance and had high positive loadings for the edge and slope of hearing loss in both ears, and had high negative loadings for the degree of hearing loss in both ears. The second factor explained 22% of the variance and had high positive loadings for the remaining audiometric variable (frequency of the worst hearing level) in both ears. The third explained 12% of the variance and had high positive loadings for the slope and degree of hearing loss in the two ears.

**Table 2:** Details about the loadings of each of the eight principal components onto the original audiometric variables. Components are statistical constructs, but the individual loadings indicate the ‘meaning’ of each one. For example, principal component 1 most strongly represents the edge and the slope of the hearing loss.

	Principal components							
Audiometric variables	1	2	3	4	5	6	7	8
Edge of HL in the steeper ear	0.731	0.245	0.110	0.458	−0.427	−0.027	0.000	0.001
Edge of HL in the less-steep ear	0.808	0.088	0.191	0.274	0.455	−0.040	−0.140	−0.015
Slope of HL in the steeper ear	0.746	−0.208	0.439	−0.338	−0.172	0.216	−0.130	−0.004
Slope of HL in the less-steep ear	0.825	−0.314	0.363	−0.073	0.104	−0.109	0.245	0.026
Degree of HL in the steeper ear	−0.830	0.262	0.465	0.109	0.039	0.032	−0.036	0.100
Degree of HL in the less-steep ear	−0.797	0.232	0.541	0.072	0.009	0.026	0.064	−0.094
Frequency of the worst HL in the steeper ear	0.294	0.851	0.070	−0.303	−0.060	−0.294	−0.047	−0.001
Frequency of the worst HL in the less-steep ear	0.375	0.842	−0.173	−0.020	0.119	0.306	0.115	0.006
Variance explained (%)	49.7	22.1	11.5	6.4	5.6	3.0	1.5	0.2
Eigenvalue	3.0	1.8	0.9	0.5	0.4	0.2	0.1	0.0

The multiple regression model specified the dominant tinnitus pitch as the criterion variable with the three selected principal components as predictor variables. Given the evidence that tinnitus bandwidth plays an important role in modulating the relationship between tinnitus pitch and audiometric profile, bandwidth categories were included in the model as a fixed between-subjects factor. The interactions between each of the three principal components were also included as predictor variables in the model. The model was not successful in predicting tinnitus pitch across the whole cohort (F[11] = 1.022, p > .05). If only people with a tonal tinnitus experience a tinnitus pitch that is determined by their audiometric edge, then including data from people whose tinnitus has a moderate or broad bandwidth might weaken the overall relationship, despite including it as a moderator variable in the model. However, analysing only those data for people with a narrow tinnitus bandwidth would be too lacking in statistical power. In the discussion we propose a way to deal with this issue.

## Discussion

The present study set out to explore the relationship between audiometric profile and tinnitus pitch in a large cohort of participants. The data broadly support the view that tinnitus is associated with the degree of hearing loss, and that the tinnitus pitch more typically falls within the region of hearing loss than at (or close to) the edge of hearing loss. No further systematic relationships were found between audiometric variables and tinnitus pitch when all the participants were considered as a single group. The strongest positive correlations emerged when the analysis was limited to the subgroup reporting a narrow tinnitus bandwidth. For these subj ects, their dominant tinnitus pitch was about an octave above the edge in the area of hearing loss - a result that is inconsistent with the tonotopic expansion model of tinnitus which predicts that the pitch should *correspond* to the edge frequency. Although significant, these correlation coefficients had large confidence intervals suggesting some degree of uncertainty about whether the observed r value truly represents the population. We suggest that more substantial evidence to refute or support alternative neurophysiological models for tinnitus will be best obtained by using appropriate *a priori* inclusion criteria (namely, participants with a narrowband tinnitus), and to recruit a large sample of such participants. This would generate a reasonably homogenous sample of people with tinnitus in sufficient numbers to yield a high degree of statistical certainty in the outcome.

### Implications of the pitch matching procedure

Our data demonstrated some discrepancy between the participant's classification of their tinnitus spectral properties and their subsequent psychoacoustical ratings of tinnitus bandwidth (shown in Figure 3). Two possible reasons for this discrepancy are proposed. One explanation concerns the reliability of the measures. Perceptual matching (e.g. bracketing method) is widely known to be a difficult procedure (be it for pitch or loudness, Andersson et al, 2005), and as a consequence results tend to be somewhat unreliable (Penner, 1983; Henry & Meikle, 2000). We are currently collecting data to address the test-retest reliability of the tinnitus spectrum measured using the Tinnitus Tester. Nevertheless, it has already been shown that group-averaged spectra considerably overlap when participants are measured twice (Roberts et al, 2008). The second explanation relates to the validity of the subjective classification (tonal, ringing, or hissing) especially when the timbre of the reference sounds are not representative of the individual's tinnitus percept. Top-down influences could be a further contributing factor that affects this validity of the classification (tonal, ringing, or hissing) for people who experience a complex percept. There is a wealth of evidence that patients’ perception of tinnitus can be influenced by psychological factors such as stress and anxiety (e.g. Fagelson, 2010, Andersson et al, 2005) and a heightened involvement of the emotional centres of the brain has also been indicated in patients with tinnitus (Adjamian et al, 2009). Psychological factors can influence the way in which the individual sensation of tinnitus is perceived and so we suggest that such top-down influences can make any classification of the tinnitus sound unreliable, especially when we force patients to choose from only three options (tonal, ringing, and hissing) to describe their tinnitus percept.

### Comparison with other studies

**Figure 6:**
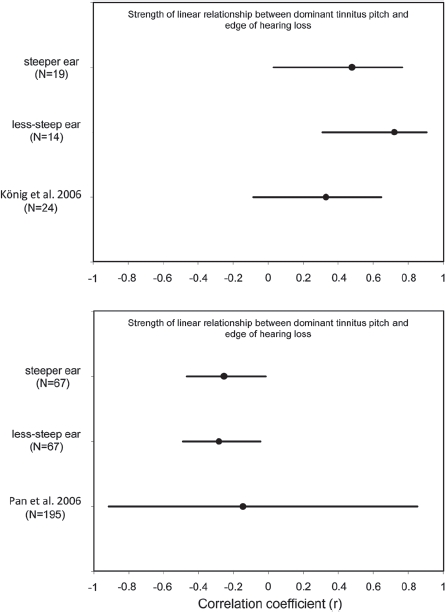
Comparison of Pearson's correlation coefficients and confidence intervals across studies. The top panel shows the strength of the relationship between dominant tinnitus pitch and the edge of hearing loss in patients reporting a narrow tinnitus bandwidth ('tonal'). The bottom panel shows the strength of the relationship between the dominant and the degree of hearing loss in the whole patient cohort. Our present data are reported split according to their steeper and less-steep deafened ear.

Given that correlation analyses are more useful for data exploration than for hypothesis testing, it is informative to seek convergent support for our main findings through comparison with other studies. In this section, we therefore compare our results with those of König et al (2006) who reported an association between dominant tinnitus pitch and edge of hearing loss in people with tonal tinnitus and with those of Pan et al (2009) who reported an association between tinnitus pitch and degree of hearing loss. Before we consider the main findings, we highlight several issues about how correlation results are reported. While many authors depend greatly on the statistical significance level to interpret the results, in many cases, even though the correlation coefficient (r) is significant, the chosen variable can account for only a very small percentage of the variability (r^2^). Confidence intervals on the other hand give us information about likely value of the correlation coefficient (r) in the population. If two samples have confidence intervals that do not overlap it can indicate that samples were taken from different populations. If they do overlap, the sample was probably taken from the same population. We can apply this logic to compare our results with those of other studies even though the actual correlation coefficients obtained were very different. A positive finding from our correlation analysis was a significant relationship between dominant tinnitus pitch and edge of hearing loss in people with narrowest tinnitus bandwidth, somewhat consistent with König et al (2006). For König and colleagues, the likely value of r fell between — 0.08 and 0.65, while for our data it fell between 0.03 and 0.77 (steeper ear) and 0.31 and 0.91 (less-steep ear) (see Figure 6). We note that these confidence limits are rather broad which means that there is weak confidence in the reliability of the correlation values. For our subjects with narrow tinnitus bandwidth, 23 and 52% of variance in dominant tinnitus pitch could be accounted for by the edge of hearing loss (for the steeper and less-steep ears, respectively), while for König and colleagues, this association accounted for only 11% of the variance.

An important issue for future experimental design concerns sample size. To reliably estimate the strength of an association we need to obtain narrower confidence intervals. Based on the above observations, we therefore recommend that future studies should aim to reduce the confidence intervals by at least half. Halving the confidence intervals can be achieved by quadrupling the sample size. Hence, a sample of 100 tinnitus subjects is needed to reliably evaluate the association between tinnitus pitch and the edge of hearing loss.

Simply increasing the sample size is probably unlikely to be sufficient for improving reliability, since it does not address the issue of heterogeneity. This point can be illustrated by considering the strength of the association between tinnitus pitch and degree of hearing loss, comparing our data (n = 67) with those of Pan et al (2009) (n = 195). Our confidence intervals were much narrower than those obtained by Pan and colleagues (Figure 6), even though a larger cohort of patients was tested. Hence, not only should the sample size be increased in order to obtain reliable results, but also careful selection of patients is crucial in order to reduce variability. To reliably evaluate the association between tinnitus pitch and the edge of hearing loss, we specifically recommend that a sample of 100 subjects with a narrow tinnitus bandwidth is needed.

## Supplementary material available online

### Appendix

The supplementary appendix can be found online at: http://www.informahealthcare.com/doi/abs/10.3109/14992027.2010.551221.
