# Supplementary material for: Re-examining the relationship between audiometric profile and tinnitus pitch
Source: Int J Audiol. 2011 Mar 9;50(5):303–12. doi: 10.3109/14992027.2010.551221 (PMC3082165; doi:10.3109/14992027.2010.551221)
Supplement: Supplementary file 1 [file tija50-303-SD1.pdf]

Supplementary material for Sereda M, Hall DA, Bosnyak DJ, Edmondson-Jones M, Roberts LE, Adjamian P, Palmer AR. 2011. Re-examining the relationship between audiometric profile and tinnitus pitch. *Int J Audiol*, 50, 303–312.

Appendix

Correlation between audiometric variables (edge of the hearing loss, slope of the hearing loss, degree of hearing loss, and the frequency of the worst hearing level, for the two ears) were highly inter-correlated; to access, go to http://www.informahealthcare.com/doi/abs/10.3109/14992027.2010.551221.

Appendix 1. Correlations between the different audiometric variables (audiometric edge, slope, degree of hearing loss, frequency of the worst hearing level) for the steeper and less steep ears. Values in bold indicate a significant relationship ($p < .002$, corrected for multiple comparisons).

	Edge of HL in the less- steep ear	*Slope of HL in the steeper ear*	*Slope of HL in the less-steep ear*	*Degree of HL in the steeper ear*	*Degree of HL in the less-steep ear*	*Frequency of the worst HL in the steeper ear*	*Frequency of the worst HL in the less-steep ear*
Edge of HL in the steeper ear	**r .565 p .001**	**r .532 p .001**	r -.128 p .328	**r -.590 p .001**	**r .567 p .001**	**r .390 p .002**	**r .415 p .001**
Edge of HL in the less-steep ear		**r .507 p .001**	**r .705 p .001**	**r -.509 p .001**	**r -.505 p .001**	r .234 p .118	r .364 p .013
Slope of HL in the steeper ear			**r .383 p .001**	r -.137 p .270	r -.077 p .534	**r .413 p .001**	**r .432 p .001**
Slope of HL in the less-steep ear				**r .369 p .002**	**r .503 p .001**	r .333 p .006	r .330 p .007
Degree of HL in the steeper ear					**r .952 p .001**	r .249 p .042	r .062 p .620
Degree of HL in the less-steep ear						r .325 p .007	r .07 p .566
Frequency of the worst HL in the steeper ear							**r .546 p .001**
